# The interplay between adipose-derived stem cells and bladder cancer cells

**DOI:** 10.1038/s41598-018-33397-9

**Published:** 2018-10-11

**Authors:** Malgorzata Maj, Anna Kokocha, Anna Bajek, Tomasz Drewa

**Affiliations:** Chair of Urology, Department of Tissue Engineering, Collegium Medicum, Nicolaus Copernicus University, Karlowicza 24, 85–092 Bydgoszcz, Poland

## Abstract

Tissue engineering approaches offer alternative strategies for urinary diversion after radical cystectomy. Possible triggering of cancer recurrence remains, however, a significant concern in the application of stem-cell based therapies for oncological patients. Soluble mediators secreted by stem cells induce tissue remodelling effects, but may also promote cancer cells growth and metastasis. We observed a substantial increase in the concentration of IL-6 and IL-8 in the secretome of adipose-derived stem cells (ASCs) co-cultured with bladder cancer cells. Concentrations of GM-CSF, MCP-1 and RANTES were also elevated. Bioactive molecules produced by ASCs increased the viability of 5637 and HT-1376 cells by respectively 15.4% and 10.4% (p < 0.0001). A trend in reduction of adhesion to ECM components was also noted, even though no differences in β-catenin expression were detected. When HT-1376 cells were co-cultured with ASCs their migration and invasion increased by 24.5% (p < 0.0002) and 18.2% (p < 0.002). Expression of p-ERK1/2 increased in 5637 cells (2.2-fold; p < 0.001) and p-AKT in HB-CLS-1 cells (2.0-fold; p < 0.001). Our results confirm that ASCs crosstalk with bladder cancer cells *in vitro* what influences their proliferation and invasive properties. Since ASCs tropism to tumour microenvironment is well documented their application towards post-oncologic reconstruction should be approached with caution.

## Introduction

Bladder cancer (BC) is the fourth most common cancer worldwide. The highest incidence rates are observed in Southern and Western Europe, Northern America, and Western Asia^1,2^. Even though mortality rates have been decreasing in recent years, BC remains substantial health burden due to high recurrence rates^3,4^. Radical cystectomy (RC) is considered the gold standard for the treatment of muscle-invasive and high-risk non-muscle-invasive BC with minor or no significant differences in oncological outcomes when comparing open RC with laparoscopic and robot-assisted RC^5–7^. The procedure involves removal of the entire bladder, lymph nodes, part of the urethra, and nearby organs that may contain cancer cells. Still, patients after RC remain at risk of BC recurrence with remaining urethra as a common recurrence site^8^.

Both continent and incontinent diversions are available for bladder replacement after RC. Due to significant problems associated with the use of gastrointestinal segments for bladder augmentation, new methods for urinary tract reconstruction are being sought^9,10^. Most of these methods use cell-seeded matrices to build tissue-engineered tubular grafts^11,12^. New, biologically derived scaffolds seeded with autologous cells for bladder wall substitution are also investigated^13–15^. Several cell types, e.g. bladder epithelial cells, smooth muscle cells, adipose-derived stem cells, or urine-derived stem cells are used for seeding onto scaffolds to promote tissue regeneration. Still, most techniques for scaffolds production employ autologous adipose-derived stem cells (ASCs)^16–18^.

ASCs are considered as the most suitable source of cells for stem cell-based therapies mainly because they can be harvested in large quantities using minimally invasive procedures^19–21^. It has been shown that ASCs secrete a wide variety of soluble mediators that promote morphological regeneration and functional restoration of bladder defects^22–24^. Possible triggering of cancer recurrence during remission remains, however, a significant concern in the application of stem cell-based therapies for cancer patients. It is suggested that paracrine factors secreted by locally delivered ASCs may induce activation of persisting tumour-initiating cells^25^. Despite intensive investigation, the influence of ASCs on cancer progression remains mostly unclear.

Previously we showed that conditioned medium form ASCs culture (ASC-CM) reduces bladder cancer cells viability and increases their resistance to ciprofloxacin, an antibiotic used to treat many bacterial infections, including urinary tract infections^26^. To gain further insight into the nature of interactions between ASCs and bladder cancer cells we co-cultured both cell types in a transwell system that prevents passage of cells but allows bidirectional transport of soluble factors. Then we analysed the composition of ASC-CM, quantified changes in viability, proliferation, adhesion, and migration of cancer cells, and examined activation of critical pro-survival pathways that are known for promoting cell growth, regulating apoptosis, chemotherapeutic drug resistance, and cellular senescence.

## Results

### Multiplex protein analysis

Qualitative and quantitative analysis of ASC-CM composition is essential in order to identify key players influencing the biological activities of these cells. When ASCs were co-cultured with human primary bladder carcinoma cell lines (Table 1) a strong increase in protein concentration was observed for IL-6 (from 23-fold to 3.9-fold depending on the cell line) and for IL-8 (from 16.1-fold to 10.3-fold). A moderate increase in the concentration of GM-CSF (from 3.6-fold to 2.3-fold), MCP-1 (from 2.3-fold to 1.7-fold), and RANTES (from 4.5-fold to 1.5-fold) were also noted. No significant changes in the level of IL-1B, TNF-α, and TGF-β1 were observed in co-culture with cancer cells in comparison to monoculture. The presence of IL-1A, IL-4, IL-10, and IFN-γ in ASC-CM could not be detected.

**Table 1:** Changes in protein levels in ASC-CM after co-culture with human bladder carcinoma cell lines.

Protein	5637		HB-CLS-1		HT-1376	
	pg/ml	fold change	pg/ml	fold change	pg/ml	fold change
IL-1B	0.60 (0.09)	1.5 ↑	n.d.	n.d.	n.d.	n.d.
IL-6	34.75 (9.96)	23.0 ↑	22.20 (3.32)	14.7 ↑	5.89 (1.43)	3.9 ↑
IL-8	25.93 (3.00)	16.1 ↑	21.21 (5.58)	13.2 ↑	16.50 (2.89)	10.3 ↑
TNF-α	1.86 (0.71)	1.2 ↑	n.d.	n.d.	1.76 (0.43)	1.2 ↑
GM-CSF	5.51 (1.45)	3.6 ↑	3.5 (0.87)	2.3 ↑	n.d.	n.d.
MCP-1	101.82 (14.78)	2.3 ↑	90.64 (10.93)	2.0 ↑	87.79 (6.09)	1.7 ↑
TGF-β1	6.22 (3.54)	1.2 ↑	n.d.	n.d.	6.45 (2.11)	1.3 ↑
RANTES	1.64 (0.56)	4.5 ↑	0.90 (0.21)	2.5 ↑	0.55 (0.09)	1.5 ↑

Standard deviation is given in brackets. n.d. –not detectable.

### Cell morphology

**Figure 1:**
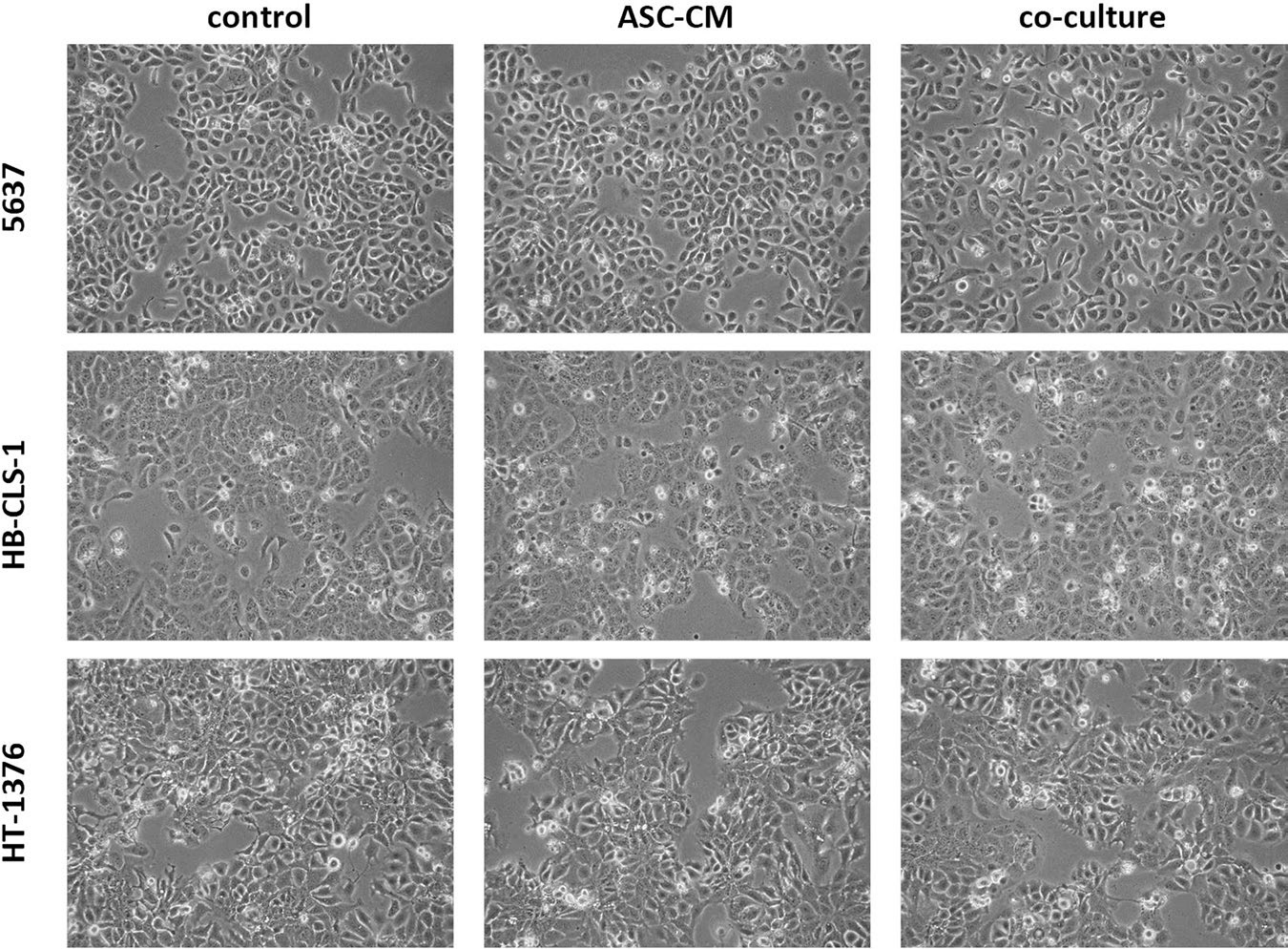
Morphology of bladder carcinoma cell lines cultured in ASC-CM or in co-culture with ASCs. Magnification ×100.

The culture of carcinoma cell lines in ASC-CM or co-culture with ASCs did not influence the morphology of cancer cells (Fig. 1). No signs of deterioration, e.g. granularity around the nucleus, detachment of the cells from the growth surface or cytoplasmic vacuolation were observed. Microscopic images were further used for quantitative analysis (cellSens Software, Olympus). No changes in shape, perimeter, as well as cell and nuclear volumes, were detected in comparison to monoculture (data not shown).

### Proliferation

**Figure 2:**
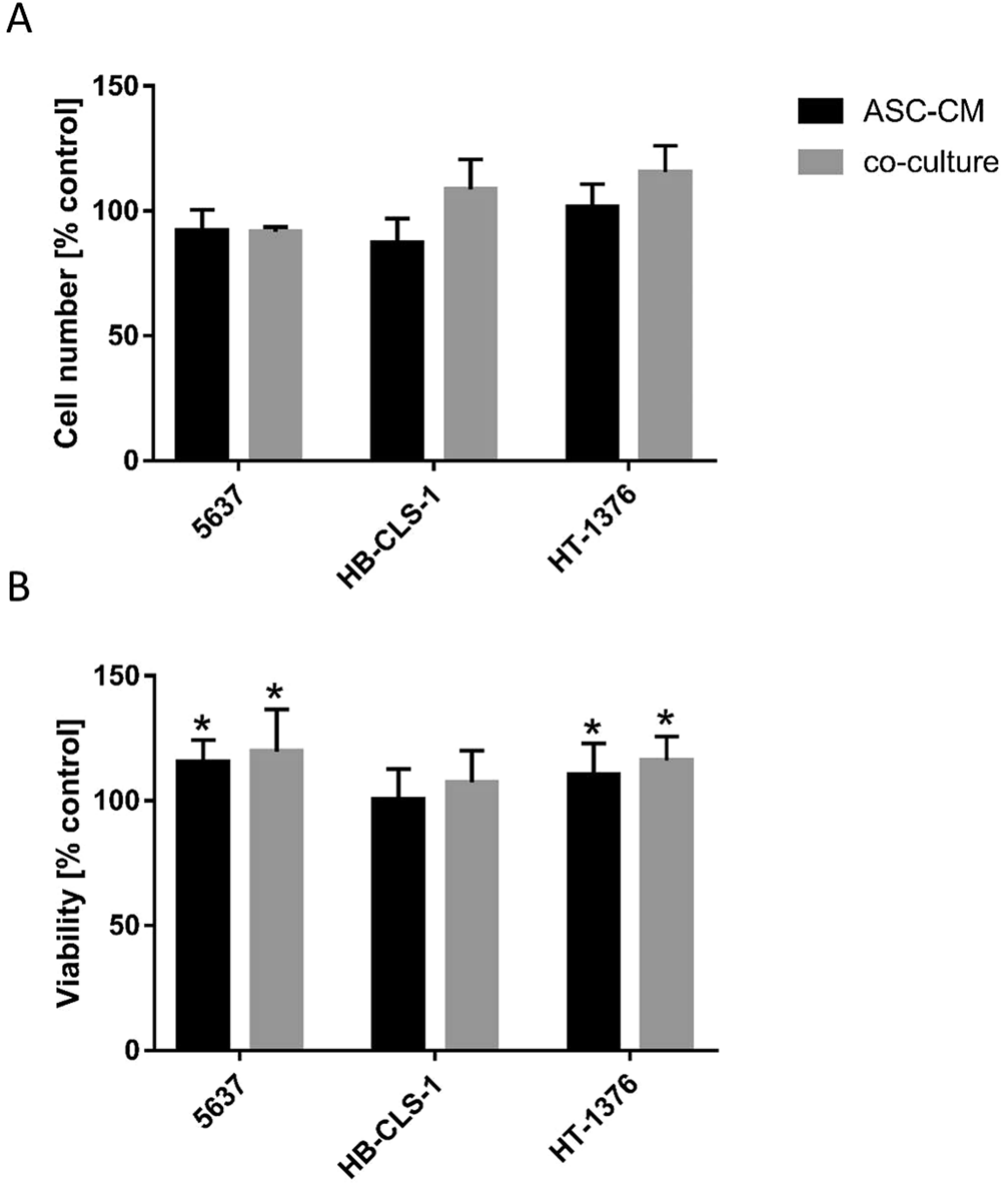
Proliferation and viability of carcinoma cells cultured in ASC-CM and in co-culture with ASCs. (**A**) Soluble mediators secreted by stem cells increased the number of HB-CLS-1 and HT-1376 cells by respectively 8.5% and 15.5% compared to control. Interestingly, this effect was only observed when cells were co-cultured with ASCs not when they were cultured in ASC-CM. Bars represent standard deviation. (**B**) Conditioned medium from ASCs increased the viability of 5637 and HT-1376 cells by respectively 15.4% and 10.4% compared to control. ASC-CM did not influence the metabolic activity of HB-CLS-1 cells. Similarly, co-culture with stem cells increased the viability of 5637 and HT-1376 cells (19,7% and 16.1%), but not of HB-CLS-1 cells. Bars represent standard deviation; p < 0.05.

Co-culture with ASCs increased the number of HB-CLS-1 and HT-1376 cells by respectively 8.5% and 15.5% compared to control (Fig. 2A). These changes were not, however, statistically significant (p > 0.05). No differences in the number of 5637 cells were observed after co-culture with ASCs. Culture in ASC-CM did not influence the proliferation rate in any of the bladder carcinoma cell lines. No statistically significant differences were observed between the culture in ASC-CM and co-culture with cancer cells for any of the bladder carcinoma cell lines.

### Viability

The metabolic activity of 5637 and HT-1376 cells after culture in ASC-CM was increased by respectively 15.4% (p < 0.0001) and 10.4% (p < 0.0001) (Fig. 2B). Co-culture with ASCs increased the viability of both bladder carcinoma cell lines by respectively 19.7% (p < 0.001) and 16.1% (p < 0.0001). No changes were, however, observed for HB-CLS-1 cells. Interestingly, no statistically significant differences were noted between the viability of cancer cells cultured in conditioned medium and co-cultured with stem cells.

### Cell cycle

**Figure 3:**
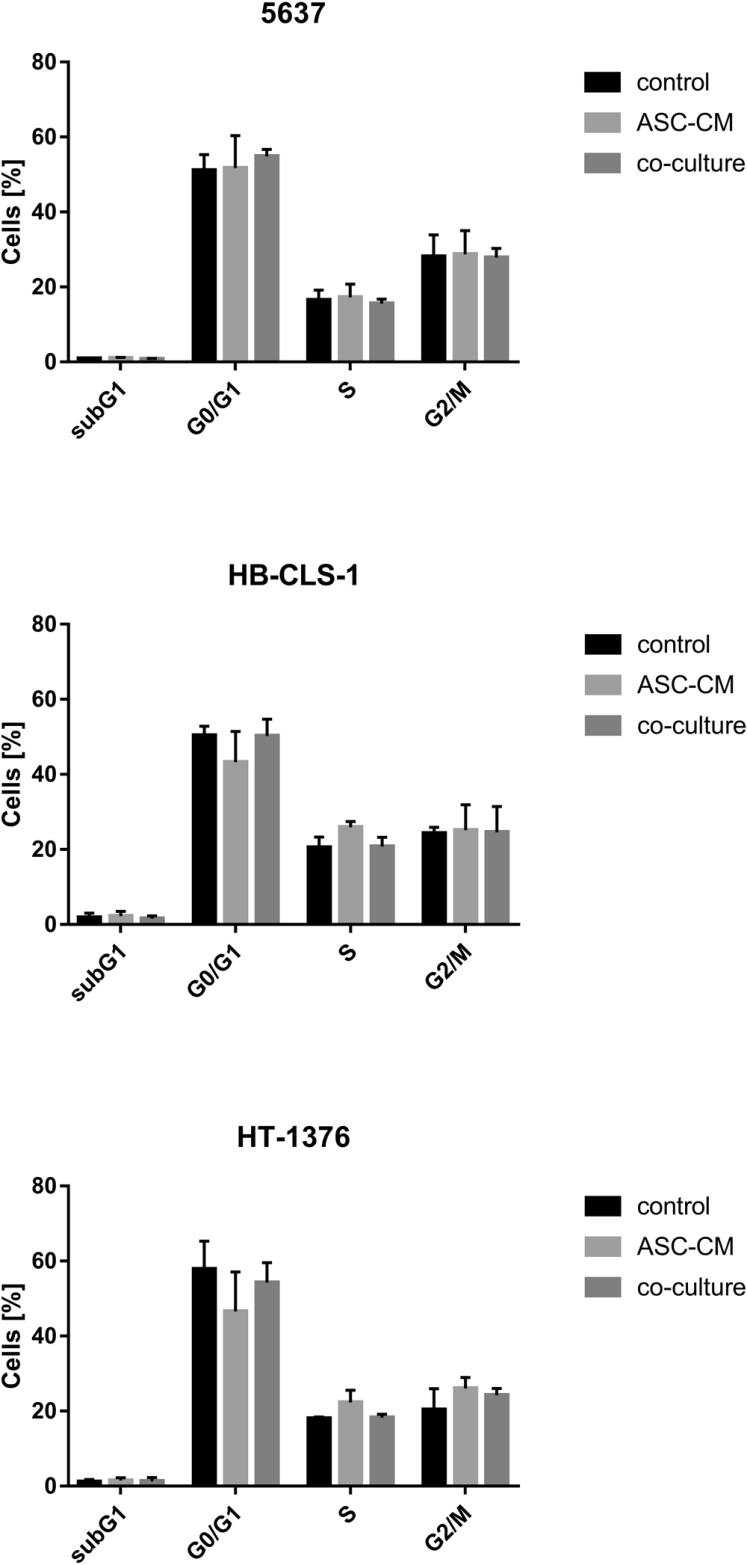
Cell cycle perturbations in carcinoma cells cultured in ASC-CM and in co-culture with ASCs. Culture in ASC-CM reduced the proportion of HB-CLS-1 and HT-1376 cells in G0/G1 phase by 7.2% and 11.3% and increased the proportion of cells in S phase by respective 5.4% and 4.3%. In contrast to incubation in conditioned medium, co-culture with ASCs did not influence the distribution of cells in subsequent phases of the cell cycle. No changes were observed for 5637 cell line. Bars represent standard deviation.

The proportion of HB-CLS-1 and HT-1376 cells in G0/G1 phase was reduced by respectively 7.2% and 11.3% after incubation with ASC-CM (Fig. 3). At the same time, the proportion of cancer cells in S phase was increased by respectively 5.4% and 4.3%. These changes were not, however, statistically significant (p > 0.05). Co-culture with stem cells did not influence the cell cycle of both bladder cancer cell lines. Similarly, no changes in the distribution of 5637 cells in subsequent phases of the cell cycle were noted after incubation in ASC-CM and after co-culture with ASCs.

### Apoptosis

**Figure 4:**
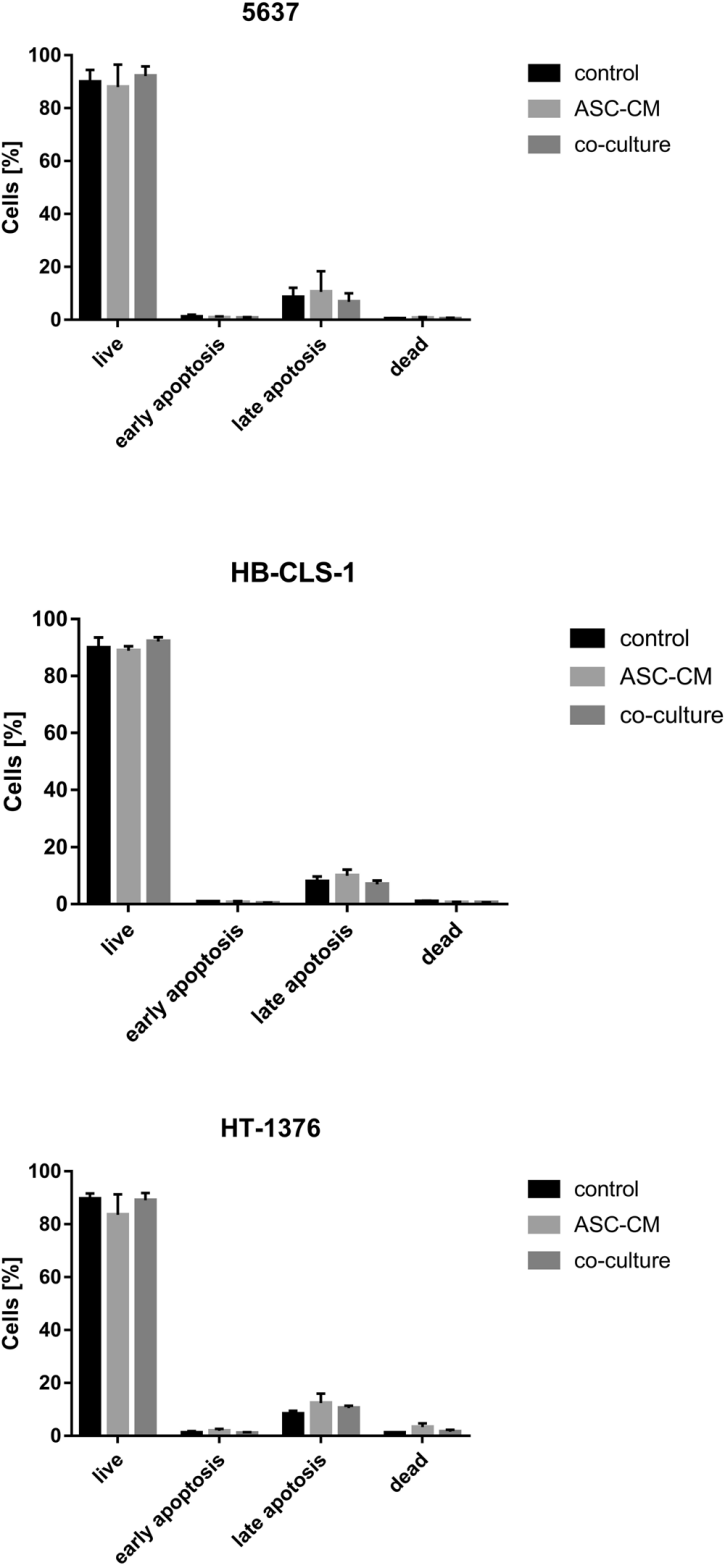
Membrane phospholipid redistribution in carcinoma cells cultured in ASC-CM and in co-culture with ASCs. Annexin V positive/propidium iodide negative cells were classified as early-stage apoptotic cells, and double-positive cells were classified as late-stage apoptotic cells. Culture in ASC-CM did not induce changes in the membrane asymmetry. Co-culture with ASCs reduced the number of live HT-1376 cells by 6.1% and increased the number of late apoptotic cells by 4.0%. Bars represent standard deviation.

Loss of plasma membrane asymmetry is one of the earliest features of apoptotic cells. Co-culture with ASCs did not induce translocation of phosphatidylserine from the inner to the outer leaflet of the plasma membrane of cancer cells (Fig. 4). A minor reduction in the number of live cells (6.1%) with a simultaneous increase in the number of late-apoptotic cells (4.0%) was observed for HT-1376 cell line after incubation in ASC-CM. These changes were not, however, statistically significant (p > 0.05).

### ECM adhesion

**Figure 5:**
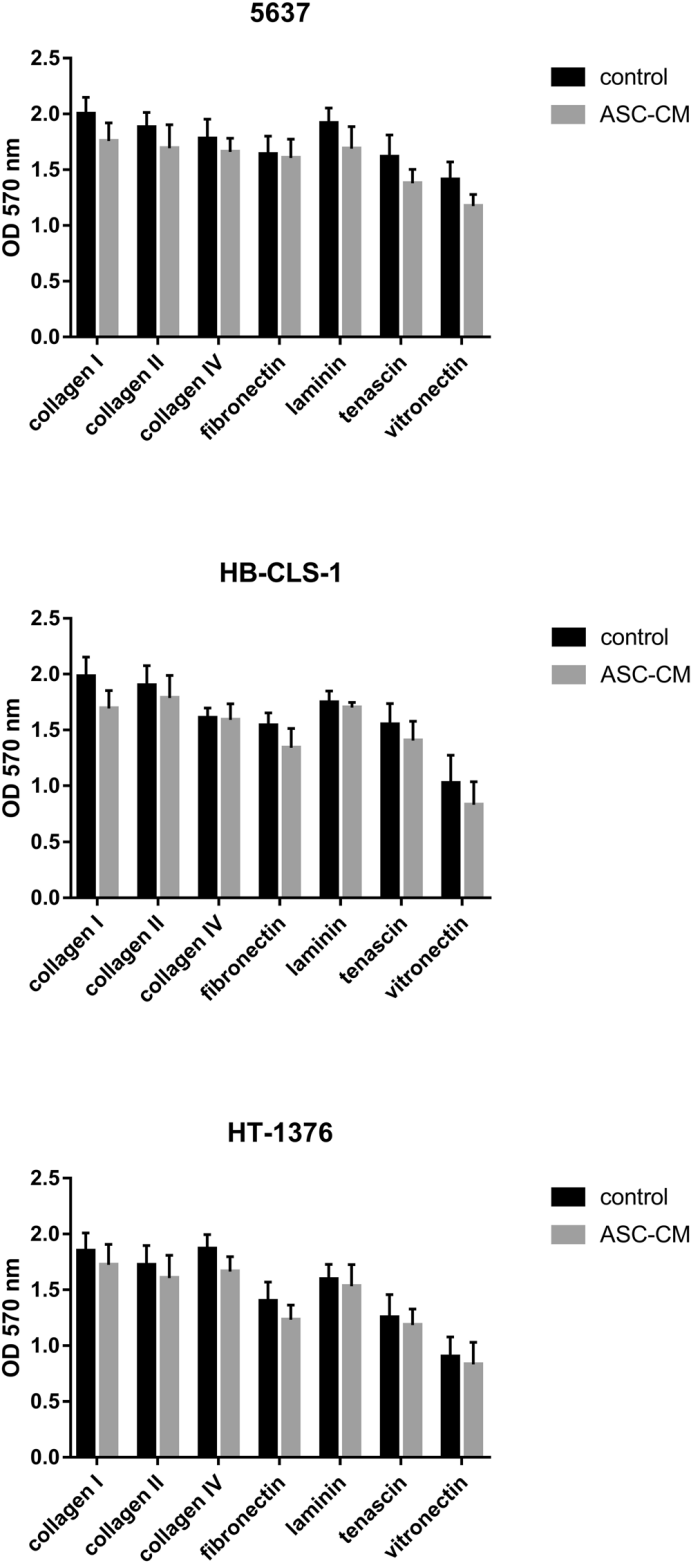
Adhesion of bladder carcinoma cells to ECM proteins after culture in ASC-CM. Even though observed changes did not reach statistical significance a trend in the reduction of cell adhesion to ECM components (collagen I, collagen II, collagen IV, fibronectin, laminin, tenascin, vitronectin) could be noted. Bars represent standard deviation.

Cell adhesion plays a significant role in cellular communication and regulation, influencing migratory and invasive properties that promote cell motility. A trend in reduction of cell adhesion to all analysed ECM components (collagen I, collagen II, collagen IV, fibronectin, laminin, tenascin, vitronectin) after incubation with ASC-CM could be seen (Fig. 5). Adhesion of 5637, HB-CLS-1 and HT-1376 cells to collagen I was reduced by respectively 12.2%, 14.6% and 6.82% in comparison to control. Depending on the cell line, adhesion to collagen II was reduced by 9.0%, 5.9%, and 6.8% and to collagen IV by 6.7%, 1.1% and 10.8%. Adhesion of cancer cells to fibronectin was reduced by 2.0%, 13.0% and 11.5%, and to laminin by 11.9%, 2.6% and 3.8%. Similarly, adhesion to tenascin was reduced by 14.7%, 9.2%, and 5.5% and to vitronectin by 16.9%, 19.1%, and 7.7%. These changes were not, however, statistically significant (p > 0.05).

### Migration

**Figure 6:**
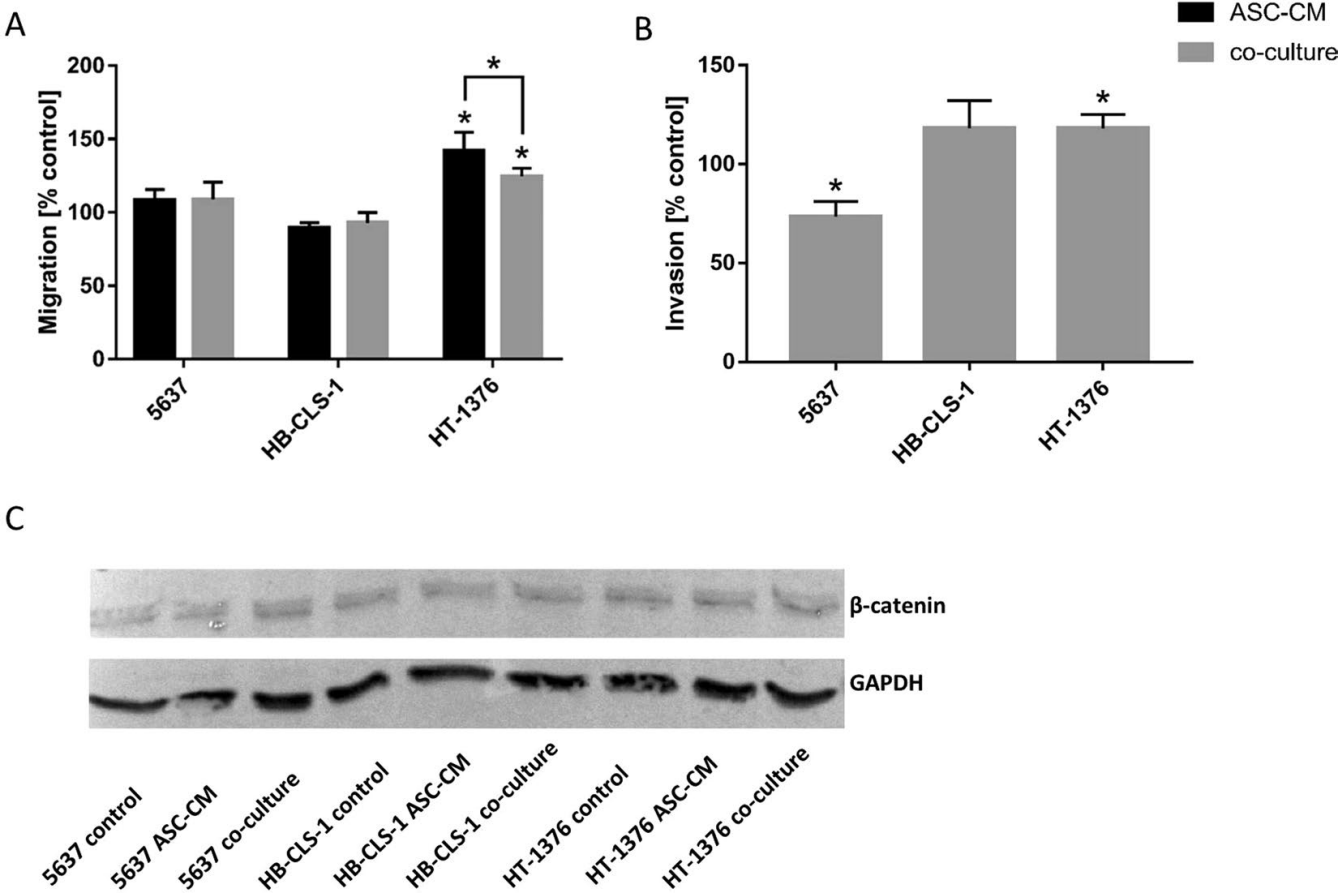
Bladder cancer cells migration and invasion. (**A**) When cultured in ASC-CM or co-cultured with ASCs HT-1376 cells showed significantly higher migration than in monoculture. Under the same conditions migration of HB-CLS-1 cells was reduced. No changes were observed for 5637 cells. (**B**) HB-CLS-1 and HT-1376 cells co-cultured with ASCs showed higher invasive properties than control cells. In turn, invasion of 5637 cells was significantly reduced. Bars represent standard deviation; p < 0.05. (**C**) Expression of B-catenin in bladder carcinoma cells did not change after culture in ASC-CM or co-culture with ASCs. Full-length blot is presented in Supplementary Figure 1.

The motility of cancer cells could be already detected in monoculture (Fig. 6A). When cultured in ASC-CM or co-cultured with ASCs the migration of HT-1376 cells was significantly increased respectively by 41.7% (p < 0.006) and 24.5% (p < 0.0002) in comparison to control. Motility of HT-1376 cells cultured in ASC-CM was significantly higher than cancer cells co-cultured with ASCs (p < 0.05). A decrease in migration of HB-CLS-1 cells was observed (10.7% and 9.2%). These changes were not, however, statistically significant (p > 0.05). No differences were noted for 5637 cells.

### Invasion

The invasion through the membrane pores could be observed in control cells (Fig. 6B). Invasive properties allowed them to digest the reconstituted basement membrane matrix of proteins derived from the Engelbreth Holm-Swarm mouse tumour, migrate through the ECM layer and cling to the bottom of the membrane. The invasion was significantly increased by 18.2% for HT-1376 cells (p < 0.002) and by 18.3% for HB-CLS-1 cells when cells were co-cultured with ASCs. Interestingly, a significant reduction of invasive properties (24.5%, p < 0.02) was noted for 5637 cells.

### B-catenin expression

B-catenin plays an essential role in the regulation of cell adhesion. Expression of B-catenin in bladder carcinoma cells cultured in ASC-CM and co-cultured with ASCs was confirmed by Western blotting (Fig. 6C). Western blot analyses were performed on whole cell lysates (20 µg per lane). No differences in signal intensities quantified by the dedicated software were detected in comparison to control (data not shown).

### AKT/ERK activation

**Figure 7:**
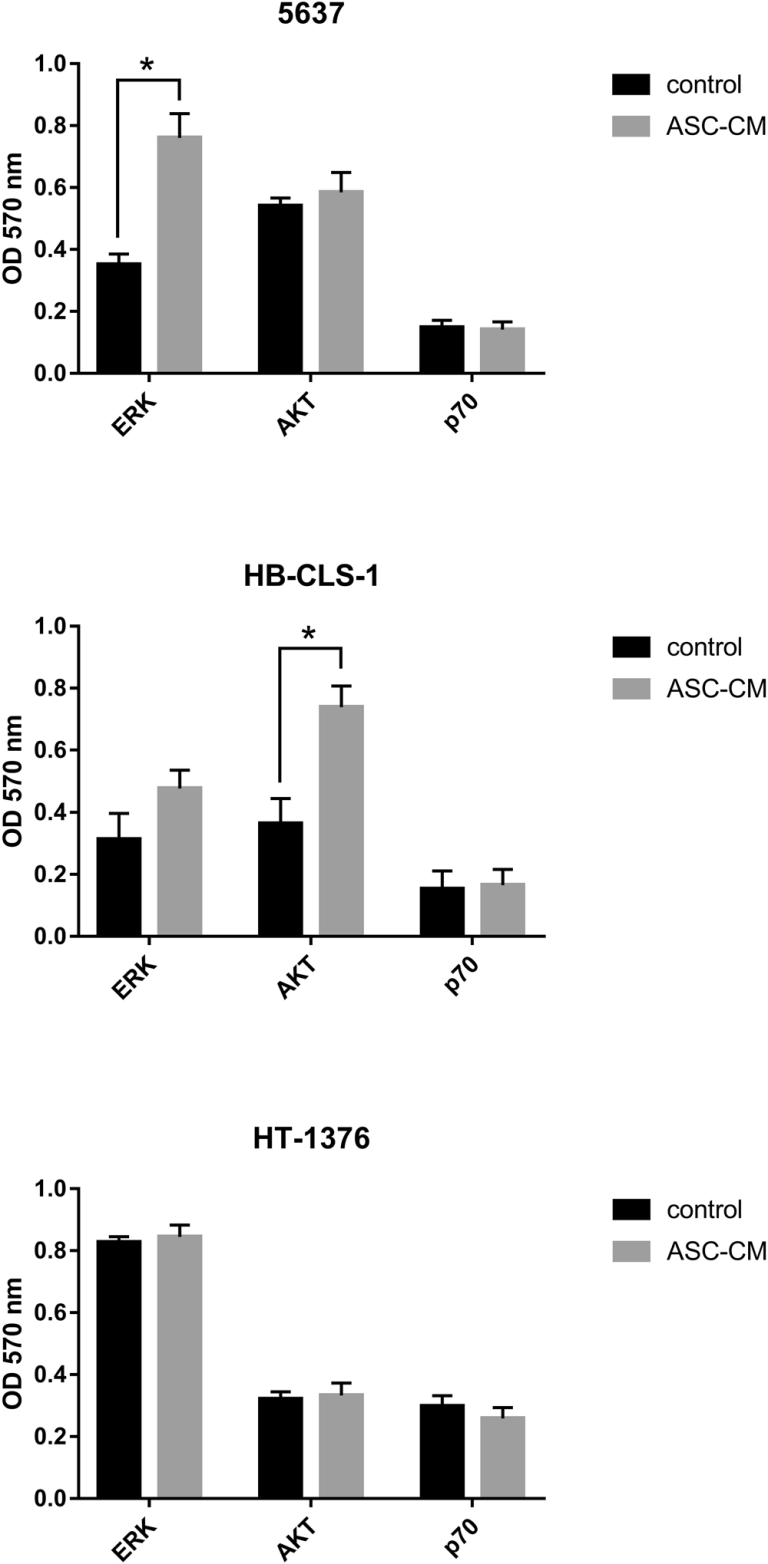
AKT/ERK/p70 S6K activation in bladder carcinoma cells cultured in ASC-CM. When cultured in ASCs secretome 5637 and HB-CLS-1 cells showed increased expression of phosphorylated ERK1/2 in comparison to control. Significant induction (2-fold) of AKT activation could also be noted in HB-CLS-1 cells. No differences in AKT/ERK activation was observed in HT-1376 cells. ASC-CM did not induce phosphorylation of p70 S6K, which activity is controlled by multiple signalling pathways, including the MAPK, PI3K, and mTOR pathways. Bars represent standard deviation; p < 0.05.

The PI3K/AKT/mTOR and MAPK signalling pathways are known for promoting cell growth, regulating apoptosis, chemotherapeutic drug resistance and cellular senescence. The InstantOne ELISA Kit used for analyses recognise several phosphorylation sites, e.g. ERK1/2/3 (Thr202/Tyr204, Thr185/Tyr187), AKT1/2/3 (Ser473) and p70 S6K (Thr389). Expression of phosphorylated ERK1/2 was significantly (p < 0.001) increased in 5637 cells cultured in ASC-CM (2.2-fold) in comparison to control (Fig. 7). Elevated expression of ERK1/2 was also observed in HB-CLS-1 cells, however, these results were not statistically significant. In turn, expression of AKT, one of the principle kinases activated by phosphoinositide 3-kinase, was significantly (p < 0.001) increased in HB-CLS-1 cells (2.0-fold). Minor increase in AKT activity was also noted in 5637 cells. No differences in AKT/ERK activation were observed in HT-1376 cells. Soluble mediators synthesised by ASCs did not influence p70 S6K activation in any of the bladder carcinoma cell lines.

## Discussion

Mesenchymal stem cells (MSCs) possess remarkable regenerative properties as they secrete a wide range of soluble mediators that induce tissue remodelling effects^27–29^. A growing body of evidence suggests that results of MSC therapy are caused by molecules, e.g. proteins, nucleic acids, lipids and extracellular vesicles, that are secreted to the extracellular environment, not to their abilities for local engrafting and differentiation. The composition of MSCs secretome is tissue-specific and changes in response to fluctuations in physiological states and pathological conditions^27,30^. Urological cancer patients, particularly those undergoing invasive surgery procedures, are considered as good candidates for regenerative therapies^31^. Possible triggering of persisting cancer cells remains, however, a significant concern in the application of autologous stem cell-based therapies during cancer remission^25,32^. Several studies reported that MSCs exhibit marked tropism for tumours^33^. Within tumour microenvironment they are thought to promote cancer cells stemness, proliferation, migration and invasion^34–37^. Through secretion of proinflammatory cytokines, chemokines and growth factors they may also contribute to epithelial to mesenchymal transition (ETM) that has been shown to play an essential role in the tumorigenic process^38^. Oh the other hand, several studies showed that ASCs induce a tumour suppressive effects *in vitro* and *in vivo*^39,40^. Oncologic follow-up at ten years after breast reconstruction with autologous fat (880 patients) showed no increased risk of local recurrence or development of new cancer^41^. Recently, this finding has been confirmed by other research groups^42,43^. However, as there has been a case of a late recurrence of osteosarcoma which occurred 13 years after the initial pathology and 18 months after a lipofiling procedure, some questions raised about the safety of MSCs usage in patients with cancer history^44^.

Giving the fact that bidirectional crosstalk between stem and cancer cells is still poorly understood, especially for urological cancers, we examined the indirect interaction between human ASCs and three human bladder carcinoma cell lines derived from primary tumours. Our results showed that co-culture of ASCs and bladder cancer cells leads to a significant change in the secreted protein levels, especially IL-6 and IL-8, proinflammatory cytokines involved in tumorigenesis (Table 1). IL-6 is one of the major cytokines present in the tumour microenvironment. Its overexpression has been reported in many types of cancers. IL-6 promotes tumour progression by regulating survival, apoptosis, proliferation, angiogenesis, and invasiveness of cancer cells. Even though emerging evidence links MSCs with metastasis, the mechanisms underlying this phenomenon are unclear^45,46^. Cortini *et al*. reported that high concentration of IL-6 in the tumour microenvironment is associated with metastatic potential of human osteosarcoma. Co-culture with bone marrow MSCs induced migratory properties through TGF-β1 and IL-6 secretion. They showed that MSCs in monoculture secreted IL-6, but when co-cultured with cancer stem cells (CSCs) derived from osteosarcoma cell lines (HOS and MG63) the levels of IL-6 significantly increased^47^. Increased secretion of IL-6 was also noted after a bone marrow, amniotic membrane, and decidua MSCs co-culture with ovarian cancer cell lines (SKOV-3 and IGROV-1) and endometrial cancer cell line (Ishikawa). IL-6 treatment decreased epithelial marker expression and increased mesenchymal markers expression, as well as MMP-2 and MMP-9 levels. A significant increase in migration and invasion of cancer cells was also noted what indicates that IL-6 facilitates metastasis and invasion by promoting EMT^48^. Similarly, Ma *et al*. reported that IL-8 and IL-6 secreted by umbilical cord MSCs activated the autocrine IL-8 and IL-6 signalling in breast cancer cell line (MCF-7) and induced CD44(+)/CD24(−) cells, which consequently promoted MCF-7 cell migration *in vitro* and metastasis *in vivo*^49^. ASCs also significantly stimulated the proliferation of MCF-7 cells in direct mixed co-cultures and transwell system, although their conditioned media displayed antiproliferative activity^50^.

Several studies reported that the synthesis of proinflammatory cytokines, notably IL-6 and IL-8, promotes activation of pro-survival pathways in cancer cells. CM from umbilical cord MSCs inhibited the expression of E-cadherin, increased the expression of N-cadherin and PCNA, as well as expression of ZEB1, a transcription factor involved in EMT through activation of the ERK pathway^51^. Chen *et al*. noted that umbilical cord MSCs activated the MEK/ERK signalling pathway in promyelocytic cells (NB4 cell line and primary acute promyelocytic leukaemia cells)^52^. Glioma-associated MSCs increased the proliferation and self-renewal of glioma stem cells *in vitro* and enhanced their tumorigenicity and mesenchymal features *in vivo* through the IL-6/gp130/STAT3 pathway activation^53^. It was also shown that bone marrow MSCs stimulated the proliferation invasion, survival, tumorigenicity and migration of colorectal cancer cells in a paracrine manner. CM downregulated the levels of phosphorylated AMPK, what indicates that MSCs promote the progression of colorectal cancer through AMPK/mTOR-mediated NF-κB activation^54^. In turn, Wnt signalling was reported to influence prostate cancer cell lines (LNCAaP, PC3 and DU145) migration toward bone marrow MSC-CM^55^. We showed that ASC-CM influences activation of critical components of two pro-survival pathways, PI3K/AKT/mTOR and MAPK, both of which are regulated by Ras (Fig. 7). When cultured in ASCs secretome two bladder carcinoma cell lines (5637 and HB-CLS-1) showed increased expression of phosphorylated ERK1/2. Additionally, the culture of HB-CLS-1 cells in ASC-CM resulted in 2-fold induction of AKT phosphorylation. Surprisingly, ASC-CM did not induce phosphorylation of p70 S6K, which activity is controlled by multiple signalling pathways, including the PI3K/AKT/mTOR and MAPK signalling pathways. Activation of ERK1/2 by MSC-CM was also reported by Scherzad *et al*. They showed that proliferation of head and neck squamous cell lines cultured in MSC-CM was induced by soluble mediators secreted by MSCs, mainly IL-6^56^.

Paracrine activity is thought to play a significant role in the cell-cell communication during tumour development and progression. Wu *et al*. showed that microvesicles (MVs) derived from human umbilical cord Wharton’s jelly MSCs inhibited bladder tumour cells growth *in vitro* and *in vivo*. After 72 h incubation with MVs (200ug/ml protein), T24 cells exhibited significant growth reduction, by 48.4% in comparison to control. Moreover, dose-dependent increase in the accumulation of cells in the G0/G1 phase with simultaneous reduction of cells in the S phase was observed. They also showed an increase in AnnexinV-FITC positive cells and dramatic up-regulation of cleaved caspase 3, p-p53 and p21^57^. Other group reported that proliferation of EJ and T24 cells cultured in ASC-CM was significantly reduced in comparison to control. Yu *et al*. also showed that ASC-CM increased the number of cells in the S phase and the percentage of early and late apoptotic cells. Apoptosis induced by soluble mediators secreted by ASCs was mediated by Bcl-2 family modulation and caspase activation. Expression of p-Akt was also decreased^58^. Umbilical cord Wharton’s jelly MSCs were also shown to inhibit proliferation of PC-3 prostate cancer cells. Co-culture with MSCs induced the cleavage of caspase 9/3 and PARP, activated JNK, and attenuated phosphorylation of Akt and ERK^59^. These results are in contrary to ours. We showed that the proliferation of bladder cancer cells co-cultured with ASCs was increased in comparison to control (Fig. 2A). Similarly, the viability of cells cultured in ASC-CM and co-cultured with ASCs was significantly increased (Fig. 2B). We observed a reduction in the proportion of HB-CLS-1 and HT-1376 cells in G0/G1 phase and subsequent increase in the proportion of cells in the S phase (Fig. 3). These results were, however, not statistically significant. No significant differences in the number of AnnexinV-FITC positive cells were noted between cells cultured in ASC-CM or co-cultured with ASCs for all tested bladder carcinoma cell lines (Fig. 4). Increased proliferation of different cancer cell lines treated with MSC-CM was noted by many research groups^60–62^. Enhanced growth of DU145 prostate cancer cells, both co-cultured with bone-marrow MSCs and treated with MSC-CM, was reported by Zhang *et al*. They showed that co-cultures of MSCs with DU154 cells promoted tube formation ability of human umbilical vein endothelial cells. Moreover, MSCs exposed to DU145 environment increased expression of markers associated with neovascularisation^63^. Du and Ju *et al*. demonstrated that MVs derived from Wharton’s jelly MSCs promoted growth and aggressiveness of renal 786-0 cells both *in vitro* and *in vivo*. MVs facilitated the progression of the cell cycle from G0/G1 to S phase^64^. Those discrepancies may be caused by the fact that the secretome is cell‐specific. Moreover, MVs comprise only a part of molecules secreted to the extracellular environment, and both soluble components and MVs are capable of independent regulation of numerous biological processes^27^.

Multiple cell adhesion molecules take part in intercellular and extracellular matrix interactions with tumour-progressing cells. The cancer-associated ECM actively contributes to tumour histopathology and behaviour. We showed a trend in the reduction of cell adhesion to ECM components (Fig. 5). Subsequently, migration of HT-1376 and invasion of HB-CLS-1 and HT-1376 cells was significantly increased after culture in ASC-CM. On the other hand, the invasion of 5637 cells cultured in the same conditions was significantly decreased (Fig. 6A,B). Giving the fact that Yu *et al*. observed a decreased migration of EJ and T24 after incubation in ASC-CM its influence might be cell-line dependent^58^. McAndrews *et al*. showed that co-culture with MSCs increased directional migration of MCF7 and MDA-MB-231 breast cancer cell lines that was mediated by TGF-β and the migratory proteins Rho-associated kinase, focal adhesion kinase, and matrix metalloproteinases^65^. ASC-CM increased the migratory activity of primary breast cancer cells isolated from human donors (two out of four samples), even though it was not parallel with increased expression of cellular receptors involved in migration^66^. We did not observe any differences in the expression of β-catenin in cells cultured in ASC-CM or co-cultured with ASCs in comparison to the control (Fig. 6C). In turn, Devarajan *et al*. reported that ASC-CM induces breast cancer cells (4T1) to express mesenchymal markers, e.g. fibronectin, alpha-smooth muscle actin and vimentin. Other group showed that direct co-culture with bone-marrow MSCs increased expression of EMT-related genes, e.g. fibronectin, SPARC, and galectin 1 in colon cancer cells (KM12SM). Interestingly, their expression was not elevated in cancer cells indirectly co-cultured with MSCs^67^.

Giving the fact that ASCs isolated from adipose tissue of patients with urological cancers show growth kinetics, surface marker expression and differentiation potential similar to ASCs isolated from adipose tissue of non-oncogenic participants they may be considered as a promising material for cell-based therapies^31^. However, there is substantial evidence that multiple human cancer cell lines, including bladder cancer cells, are responsive to signals from MSCs. We showed that co-culture of ASCs with human bladder cancer cells strongly induces secretion of IL-6 and IL-8, major pro-inflammatory cytokines present in the tumour microenvironment. Soluble mediators secreted by ASCs increased the viability and invasion of cancer cells and induced phosphorylation of ERK1/2 and AKT. It confirms that crosstalk between ASCs and cancer cells is highly complex. Therefore, a better understanding of ASCs influence on biological characteristics of bladder cancer cells is of great importance before their implantation into clinical therapy.

## Conclusion

Our results support the hypothesis that through pro-inflammatory phenotype ASCs may increase proliferation, viability, and invasiveness of bladder cancer cells. Activation of pro-survival pathways known for promoting cell growth, regulating apoptosis, chemotherapeutic drug resistance and cellular senescence indicates that through the secretion of paracrine factors stem cells may influence cancer progression. Since stem cell tropism to tumour microenvironment is well documented, increased oncological risk should be taken into account when considering the clinical use of ASCs in regenerative cell-based therapies for bladder reconstruction.

## Materials and Methods

### Cell lines

Human primary bladder cancer cell lines 5637 (cat. no. HTB-9, grade II carcinoma) and HT-1376 (cat. no. CRL1472, grade III carcinoma) were purchased from American Type Culture Collection (ATCC). HB-CLS-1 cells (cat. no. 300190, grade III carcinoma) were purchased from Cell Line Service (CLS). To exclude differences due to patient-to-patient variability ASC52telo hTERT immortalised adipose-derived mesenchymal stem cells (cat. no. SCRC-4000) obtained from ATCC were used in this study. These primary cells characterise with high viability and plating efficiency. They show high (>90%) expression of CD29, CD44, CD73, CD90, CD105, CD166 and low expression (<5%) of CD14, CD19, CD34, CD45. When maintained under optimal growth conditions ASC52telo cells have been shown to be multipotent, capable of differentiating down the adipogenic, osteogenic and chondrogenic lineages.

### Cell culture

All media and supplements, if not noted separately, were purchased from Corning, USA. 5637 and HB-CLS-1 cells were routinely cultured in RPMI1640, and HT-1376 cells were cultured in EMEM. Both media were supplemented with 10% FBS. ASC52telo cells were cultured in DMEM/Ham’s F-12 supplemented with 10% FBS and 10 ng/ml bFGF (Thermo Fisher Scientific). Cells were maintained at 37 °C in a humidified atmosphere of 5% of CO_2_.

### ASC-CM preparation

ASC52telo cells were seeded in cell culture flasks at a density of 10.000 cells/cm^2^. Conditioned medium containing soluble factors secreted by stem cells was collected after 72 h incubation, centrifuged, filtered through a vacuum-driven cup device (Millipore Express PLUS, Merck Millipore) and stored at −20 °C. Before use ASC-CM was thawed and mixed with growth medium used for cancer cells culture at ratio 1:1.

### Co-culture and culture in ASC-CM

Cancer cells were seeded on multiwell plates at a density of 5.000 cells/cm^2^, and ASC52telo cells were seeded on cell culture inserts (1um PET membrane) at the same cell density. After 24 h preincubation, inserts were transferred to multiwell plates. To investigate the influence of ASC-CM on cancer cells growth medium from selected wells was exchanged for a previously prepared conditioned medium. All experiments were performed after 72 h incubation. Cancer cells cultured in standard growth medium served as a control.

### ASC-CM composition

ASC52telo cells were seeded on 6-well plates at a density of 5.000 cells/cm^2^, and cancer cells were seeded on cell culture inserts (1 um PET membrane) at the same cell density. After 24 h preincubation inserts were transferred to 6-well plates. Cells were co-cultured up to 3 days without medium change. Inserts were then removed, and medium in 6-well plates was changed. Supernatants obtained after subsequent 24 h culture were analysed in the protein assay. ASC52telo cells from monoculture served as a control and were treated like the co-cultures. The level of cytokines/chemokines (IL-1A, IL-1B, IL-4, IL-6, IL-8, IL-10, IFN-γ, TNF-α, GM-CSF, MCP-1, TGF-β1, RANTES) was profiled with the Human Mix-N-Match Multi-Analyte ELISArray Kit (Qiagen) according to the manufacturer protocol. Briefly, supernatants and Antigen Standards were transferred to the ELISArray plate. After incubation and washing, Detection Antibody was added. Excess of Detection Antibody was removed with Washing Buffer. Then Avidin-HRP was pipetted to all wells. After incubation and washing to remove excess HRP conjugate, Development Solution was added. Finally, Stop Solution was added to each well and absorbance was read at 450 nm on a microplate reader (iMark, Bio-Rad).

### Viability

Cell viability was measured with the use of the MTT Assay Kit (Abcam) according to the manufacturer protocol. Briefly, growth medium was removed, and cells were incubated with MTT Reagent for 3 h at 37 °C. After incubation, formazan crystals were dissolved in MTT Solvent. The absorbance was read at 570 nm on a microplate reader (iMark, Bio-Rad).

### Cell cycle

Cell cycle progression was measured with the use of Tali Cell Cycle Kit (Thermo Fisher Scientific) followed by flow cytometry analysis. Cells detached from the wells were suspended in PBS and fixed in ice-cold ethanol. They were kept in ethanol overnight at −20 °C and then centrifuged. The obtained cell pellet was suspended in PBS. After subsequent centrifugation, cells were suspended in the Staining Solution composed of propidium iodide, RNase A, and Triton X-100 and analysed with the use of FACSCanto II flow cytometer (Becton Dickinson). Percentage of cells in G0/G1, S and G2 phases was calculated using the BD FACS Diva software (Becton Dickinson).

### Apoptosis

Redistribution of phosphatidylserine was measured with the use of FITC Annexin V Apoptosis Detection Kit I (BD Biosciences) followed by flow cytometry analysis. Cells detached from the wells were washed in PBS and suspended in Binding Buffer. Then FITC Annexin V and PI were added. After subsequent incubation, phosphatidylserine translocation to the outer leaflet of the plasma membrane was analysed with the use of FACSCanto II flow cytometer (Becton Dickinson).

### ECM adhesion

Cell adhesion to selected components of extracellular matrix (collagen I, collagen II, collagen IV, fibronectin, laminin, tenascin, vitronectin) was measured with the use of ECM Cell Adhesion Array Kit (Merck) according to the manufacturer protocol. Cells were detached from the wells and suspended in Assay Buffer. Then they were seeded on ECM protein-coated wells. After 2 h incubation, cells were rinsed with Assay Buffer, and Cell Stain Solution was added to each well. Wells were then washed, and Extraction Buffer was added to solubilise cell-bound stain. The absorbance was read at 570 nm on a microplate reader (iMark, Bio-Rad).

### B-catenin expression

Cell lysates used for immunoblotting were prepared in RIPA buffer (Abcam). Protein concentrations were analysed using the BCA Protein Assay Kit (Thermos Fisher Scientific). 20 ug of proteins were loaded and separated by SDS-PAGE and transferred to nitrocellulose membrane (Bio-Rad). After blocking with StartingBlock TBS (Thermo Fisher Scientific), membranes were incubated with primary monoclonal antibody against β-catenin (1:250, Thermo Fisher Scientific) and GAPDH (1:700, Thermo Fisher Scientific) at 4 °C overnight, washed and incubated with HRP-conjugated secondary antibody (1:1000, Thermo Fisher) at RT for 1 h. The membranes were then washed, stained using DAB Substrate Kit (Thermo Fisher Scientific) and visualised (Gel Doc XR + Gel Documentation System, Bio-Rad). The signal from each band was quantified by dedicated software for densitometric evaluation (Image Lab Software, Bio-Rad).

### Migration

Cell migration was measured with the use of QCM Chemotaxis Cell Migration Assay (Merck) based on the Boyden chamber principle. Cells were detached from the wells and suspended in chemoattractant-free media. The cell suspension was then added to inserts (8um, PC membrane) and medium containing 10% FBS to the lower chamber. After incubation, inserts were transferred into clean wells containing Cell Stain. Cotton-tipped swabs were used to remove the non-migratory cells layer from the interior of the inserts. Then inserts were transferred to clean wells containing Extraction Buffer. After incubation, absorbance was read at 570 nm on a microplate reader (iMark, Bio-Rad).

### Invasion

Cell invasion through a basement membrane model was measured with the use of QCM ECMatrix Cell Invasion Assay (Merck). Cells were detached from the wells and suspended in chemoattractant-free media. The cell suspension was then added to inserts (8um, PC membrane, ECMatrix) and medium containing 10% FBS to the lower chamber. After incubation, ECMatrix gel and non-invading cells were removed from the interior of the inserts with cotton-tipped swabs. Inserts were then transferred into clean wells containing staining solution. Next, inserts were washed with water, air-dried and transferred to clean wells containing EXTRACTION BUFFER. After incubation, absorbance was read at 570 nm on a microplate reader (iMark, Bio-Rad).

### AKT/ERK activation

Activation of signalling pathways was measured with the use of ERK/AKT/p70 S6K Activation InstantOne ELISA Kit (Thermo Fisher Scientific) according to the manufacturer protocol. Cells were detached from the wells and suspended in HBSS containing 5% FBS. They were transferred to the InstantOne ELISA assay microplate and lysed with Cell Lysis Mix. Antibody Cocktail was then added to selected wells. After incubation, wells were washed with Washing Buffer and Detection Reagent was pipetted to all wells. Stop Solution was added to stop the reaction. The absorbance was read at 570 nm on a microplate reader (iMark, Bio-Rad).

### Statistical analysis

Results from at least three independent experiments are presented as means ± standard deviation (SD). Statistical analyses were performed using STATISTICA 13.1 (StatSoft, Poland). Comparisons between two groups were analysed by Student’s t-test, and one-way ANOVA was used for multiple comparisons. A value of p < 0.05 was considered to be statistically significant.

## Electronic supplementary material

Supplementary Figure S1
