# Supplementary material for: The interplay between adipose-derived stem cells and bladder cancer cells
Source: Sci Rep. 2018 Oct 11;8:15118. doi: 10.1038/s41598-018-33397-9 (PMC6181926; doi:10.1038/s41598-018-33397-9)
Supplement: Supplementary file 1 — Supplementary Figure S1 [file 41598_2018_33397_MOESM1_ESM.pdf]

The interplay between adipose-derived stem cells and bladder cancer cells

Maj M.*, Kokocha A., Bajek A., Drewa T.

Chair of Urology, Department of Tissue Engineering, Collegium Medicum, Nicolaus Copernicus University, Karlowicza 24, 85-092 Bydgoszcz, Poland

* - corresponding author, Malgorzata Maj, m.maj@cm.umk.pl

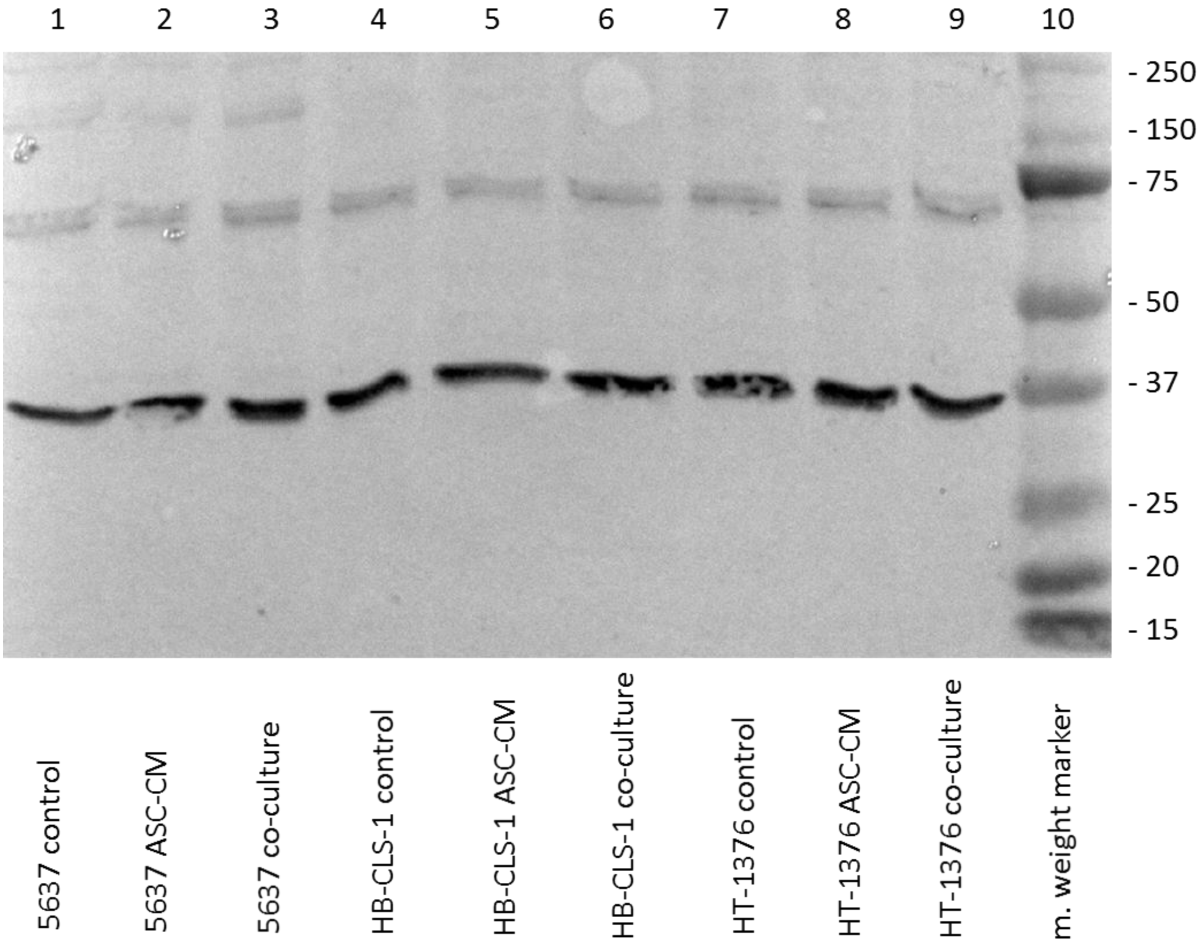

Supplementary Figure S1. *Expression of β-catenin in bladder carcinoma cell lines. Incubation in ASC-CM (lane 2, 5, 8) and co-culture with ASCs (lane 3, 6, 9) did not induce the expression of β-catenin in comparison to control (line 1, 4, 7). Blots were incubated with primary monoclonal antibody against β-catenin (Thermo Fisher Scientific) and with HRP-conjugated secondary antibody (Thermo Fisher Scientific). Positions of the relevant molecular weight markers (Precision Plus Protein Dual Color Standards, Bio-Rad) are given to the right in kDa.*
